# Genomic Diversity of *Escherichia* Isolates from Diverse Habitats

**DOI:** 10.1371/journal.pone.0047005

**Published:** 2012-10-08

**Authors:** Seungdae Oh, Sarah Buddenborg, Deborah R. Yoder-Himes, James M. Tiedje, Konstantinos T. Konstantinidis

**Affiliations:** 1 School of Civil and Environmental Engineering, Georgia Institute of Technology, Atlanta, Georgia, United States of America; 2 Center for Microbial Ecology, Michigan State University, East Lansing, Michigan, United States of America; 3 School of Biology, Georgia Institute of Technology, Atlanta, Georgia, United States of America; 4 Center for Bioinformatics and Computational Genomics, Georgia Institute of Technology, Atlanta, Georgia, United States of America; University of Vienna, Austria

## Abstract

Our understanding of the *Escherichia* genus is heavily biased toward pathogenic or commensal isolates from human or animal hosts. Recent studies have recovered *Escherichia* isolates that persist, and even grow, outside these hosts. Although the environmental isolates are typically phylogenetically distinct, they are highly related to and phenotypically indistinguishable from their human counterparts, including for the coliform test. To gain insights into the genomic diversity of *Escherichia* isolates from diverse habitats, including freshwater, soil, animal, and human sources, we carried out comparative DNA-DNA hybridizations using a multi-genome *E. coli* DNA microarray. The microarray was validated based on hybridizations with selected strains whose genome sequences were available and used to assess the frequency of microarray false positive and negative signals. Our results showed that human fecal isolates share two sets of genes (n>90) that are rarely found among environmental isolates, including genes presumably important for evading host immune mechanisms (e.g., a multi-drug transporter for acids and antimicrobials) and adhering to epithelial cells (e.g., hemolysin E and fimbrial-like adhesin protein). These results imply that environmental isolates are characterized by decreased ability to colonize host cells relative to human isolates. Our study also provides gene markers that can distinguish human isolates from those of warm-blooded animal and environmental origins, and thus can be used to more reliably assess fecal contamination in natural ecosystems.

## Introduction

*Escherichia coli* is believed to have the gastrointestinal tract of human and/or animal hosts as its preferred habitat. Accordingly, it has been traditionally thought that *E. coli* does not reproduce well outside its hosts [1]–[3], and thus, can serve as an indicator organism of fecal contamination of natural ecosystems (coliform test) [2], [4]–[6]. However, recent studies suggest that certain populations of the *Escherichia* genus, including populations conventionally classified as *E. coli,* may reproduce outside their hosts and, in fact, persist better in natural environments such as soil and water [7]–[9]. Further, the ecological and metabolic versatility of the species, reflected in the open pan-genome structure (>15,000 unique genes in the pan-genome of 61 genomes) and the high intra-species genome plasticity (i.e., nine-tenths of the pan-genome represent accessory or strain-specific genes) [10], emphasizes the functional potential of the species for adapting to a broad range of habitats and environmental perturbations, including possibly non-host associated habitats. These findings have revolutionized the traditional view of the ecology of *E. coli*.

Walk and colleagues described several clades of environmentally-adapted strains that are clearly members of the *Escherichia* genus; yet phylogenetically distinct compared to other *Escherichia* representatives (e.g., *E. fergusoni*, *E. albertii*, and *E. coli*) based on multi-locus sequence analysis (MLSA) [11]. Despite the phylogenetic differentiation, traditional phenotypic profiling based on the ability to utilize 31 carbon substrates or providing positive signal for the coliform test revealed no significant differences between the former environmental strains and human *Escherichia* representatives. Further, Ihssen and colleagues showed that environmental *E. coli* isolates, originating from raw drinking water sources, share genetic and physiological features in major important functions such as carbon utilization and stress defense with *E. coli* isolates from human and animal feces [12]. These findings have important implications for assessing contamination of natural reservoirs, which is frequently based on counting *E. coli* cells using phenotypic- and culture-based approaches [2], [4], [5]. In contrast to these previous studies, our recent study based on whole genome sequence analysis of *Escherichia* isolates from human and environmental sources identified several genes and pathways specific to each of the two sets of isolates [13]. For example, the environmental-specific genes were associated with acquisition of resources important for survival in the environment (e.g., diol utilization and lysozyme production), whereas human-specific functions were related to transport and utilization of nutrients abundant in the gut (e.g., *N*-acetylglucosamine and gluconate).

In this study, we aimed to identify new niche-specific genes for *Escherichia* isolates recovered from diverse habitats based on the analysis of twenty-seven strains, including fifteen previously uncharacterized isolates whose genomes have not been sequenced yet. We performed whole genome DNA-DNA hybridizations among isolates collected from freshwater, soil, animal, and human sources using a multi-genome *E. coli* microarray. Our comparative study revealed new gene signatures that are shared among strains recovered from human feces (enteric) and that rarely occur in environmental strains. The gene products may be needed for life in the human gastrointestinal (GI) tract, but dispensable for survival in the environment. These findings not only advance the understanding of the genetic footprint of ecological specialization in the *Escherichia* genus but also provide molecular biomarkers that can distinguish closely related, phenotypically indistinguishable enteric and environmental strains, thereby having implications for assessing and tracking fecal contamination.

## Results

### Genetic Relatedness of *Escherichia* Isolates

**Figure 1:**
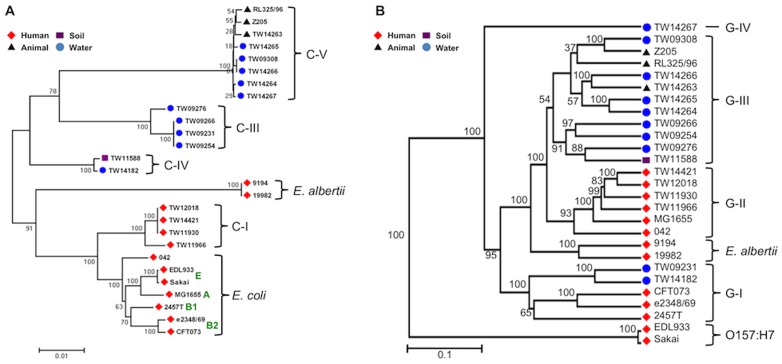
Phylogenetic (A) and gene-content (B) diversity among the *Escherichia* strains. (A) The phylogenetic analysis was carried out based on the concatenated sequence alignment of five gene sequences using the neighbor-joining algorithm in MEGA 4.0 with default settings. (B) *Escherichia* strains were clustered based on the conservation pattern of 5,978 genes by a binary system (1 = presence and 0 = absence), using the CLUSTER 3.0 software [38] with Euclidean distance. Clustering was visualized in Treeview [39]. The values on the nodes of the trees represent bootstrap support from 100 replicates in both (A) and (B).

**Figure 2:**
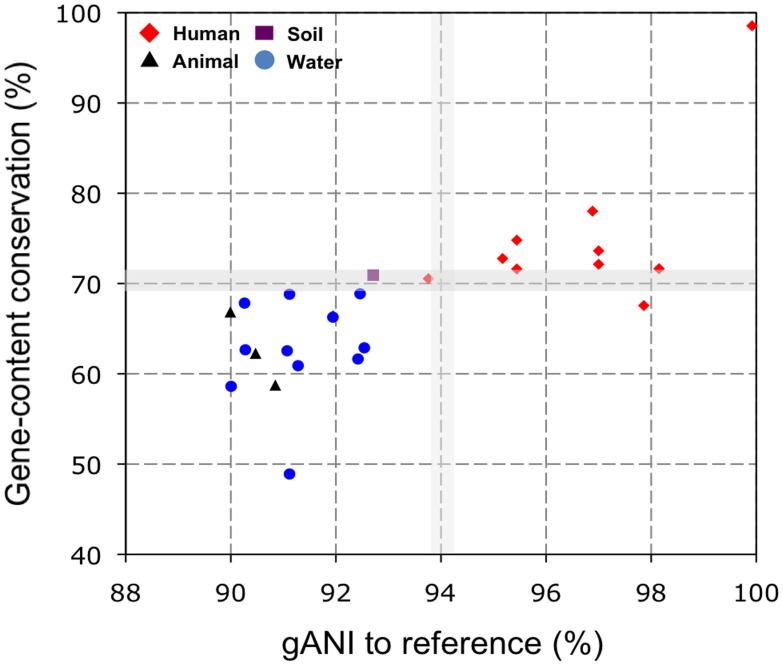
Gene content and sequence relatedness between the *Escherichia* strains and the reference strain. Each data point represents the gANI value (evolutionary relatedness) and the gene content conservation (functional relatedness) between a tester and the reference (Sakai) strains. The shared gene content (%) was normalized by the total number of the reference (Sakai) genes represented on the microarray.

Phylogenetic analysis of the concatenated sequence alignment of five MLSA genes grouped the total of 27 strains used in this study into six lineages, consistent with previous findings [11]. Three of the lineages corresponded to *E. coli, E. albertii,* and C-I strains recovered from human hosts (human strains) and the other three lineages (C-III, C-VI, and C-V) were composed of strains from environmental sources (environmental strains) or warm-blooded animal hosts (animal strains), revealing a clear phylogenetic delineation of non-human strains from the typical *E. coli* strains (Fig. 1A). Comparative genomic hybridization of the 27 strains was carried out using a spotted multi-genome *E. coli* DNA microarray based on the Qiagen (Operon) *E. coli* probe set, composed of 5,978 probes targeting most genes in three reference strains (the enterohemorrhagic Sakai and EDL933 strains and the laboratory MG1655 strain; probes for shared orthologs were designed based on the Sakai genome). Figure 2 shows functional gene content (microarray-based) and evolutionary relatedness (MLSA gene- or whole genome-based) of human and non-human strains to the reference strain (Sakai). While human strains were, not surprisingly, closely related to the reference strain, environmental and animal strains showed relatively higher divergence and had fewer genes shared with the reference strain. Notably, animal strains showed increased divergence and low gene conservation to the reference strain, comparable to that of environmental strains.

Overall, the percentage of gene content conservation of tester strains to the reference ranged from 48.9% to 98.5%, while the genome-aggregate average nucleotide identity (gANI) values, using whole genome or MLSA gene sequences, ranged from 90.0 to 99.9% (See details in Materials and Methods and Fig. S2). The human strains showed at least >67.6% gene content conservation and >93.7% gANI to the reference; the latter value approximately corresponds to the 70% DNA-DNA hybridization standard that is frequently used for species demarcation [14]. The environmental and animal strains showed <70.9% gene content conservation and <92.7% nucleotide sequence identity, indicating that these strains may represent new species compared to typical *E. coli* (human strains) based on the commonly used standards for bacterial species demarcation [15]. Our results therefore demonstrated that there is considerable difference in gene content between human and non-human strains, emphasizing that, although human strains have been studied extensively, their genomic makeup does not represent that of the total *Escherichia* population.

The results described above also indicated that gene content signatures that can distinguish human from environmental and animal strains might exist. To reveal if there are habitat-specific genes, we performed cluster analysis of microarray-based gene content patterns (e.g., gene presence/absence) for a total of 5,978 reference genes. The cluster analysis grouped strains into five groups (G-I through G-III, *E. albertii*, and O157:H7), which is mostly consistent with the phylogenetic groups but with a few notable differences (Fig. 1B). G-II was composed of enteric strains (i.e., mostly human fecal isolates): MG1655, 042, and C-I clade strains. The C-I strains are closely related but phylogenetically distinct compared to the typical *E. coli* strains such as MG1655 and enteroaggregative (EAEC) pathogen 042 (Fig. 1A). The G-III was exclusively composed of environmental and animal strains. The two O157:H7 strains (Sakai and EDL933) formed the most distinct group, which was attributable to their high evolutionary relatedness (gANI higher than 99.8%; Fig. 2), and the fact that their genomes were fully represented on the microarray [16], [17]. TW14267 clustered by its own due to the lowest number of shared genes (48.9%, Fig. 2) with the reference strain. The G-I group included two environmental strains (TW09231 and TW14182), the uropathogenic (CFT073) and the enteropathogenic (e2348/69) *E. coli* strains, and one enteroinvasive *S. flexneri* (2457T) strain. A more detailed analysis revealed that the G-I group strains lacked many prophage-associated genes (e.g., CP4-6, e14, CPZ-55, and CP4-57), which were largely shared by other groups (G-II through G-IV, except the O157:H7 group). Therefore, the absence of these prophage genes in G-I group strains rather than the presence of genes specific to G-I and pathogenicity factors accounted, at least in part, for the grouping of two environmental isolates with three human pathogens. Two *E. albertii* strains (9194 and 19982) were grouped together, which was consistent with their distinct position in the phylogenetic tree (Fig. 1A) and the distinct evolutionary path undertaken by *E. albertii* within the *Escherichia* genus [18]. G-II and G-III were the closest groups based on the conservation of gene content; yet, G-II primarily included enteric strains while G-III contained only environmental and animal ones.

### Gene Signatures of Strains Isolated from Human Sources

**Figure 3:**
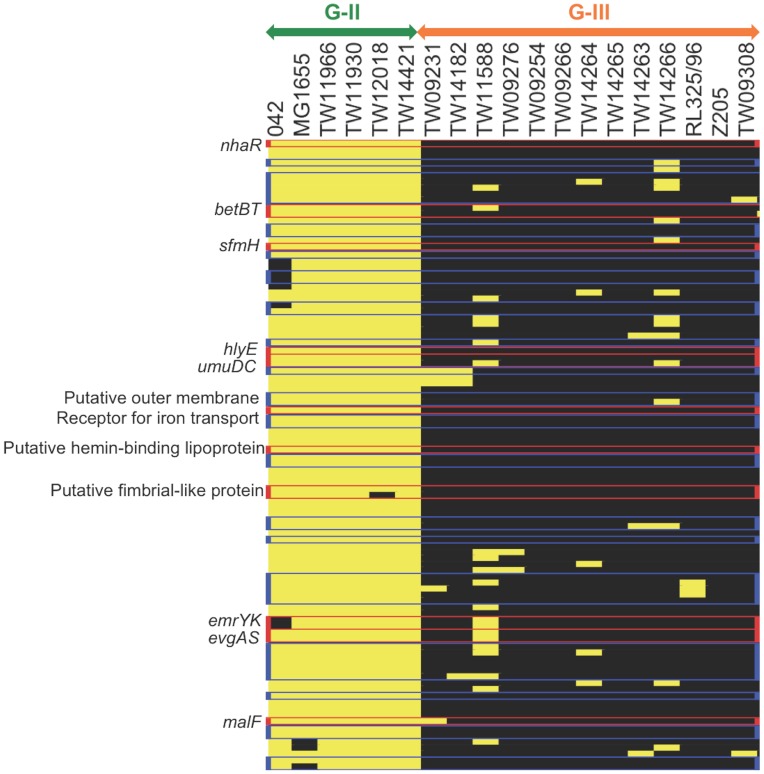
Gene signatures of human strains. The 98 genes differentially shared between G-II and G-III (p-values lower than 10^−4^ by Fisher’s exact test) are shown. Genes in blue boxes indicate hypothetical proteins and genes in red boxes are discussed in the text (associated with stress defense and adhesion). The color denotes gene presence (yellow) and absence (black).

Figure 3 shows 98 genes (*P*<10^−4^, Fisher’s exact test) conserved or highly enriched in the enteric strains of G-II and absent in the environmental and animal strains of G-III (all underlying data is available in Table S1); most of these genes were also conserved in other pathogenic strains of the GI tract such as O157:H7 (EHEC) and e2348/69 (EPEC). Although many of the genes (37 of 98 genes) encoded hypothetical proteins, the functional roles of the remaining proteins were associated with stress-defense system, cell envelope components, transport, attachment, motility, nutrient utilization, and various enzymes. The genes related to stress-defense included the *emrYK*, *evgAS*, and *umuDC*, which encode a multidrug efflux pump, a response regulator for acids and drugs, and a SOS mutagenesis protein, respectively. Two genes of the bet regulon, *betT* (high-affinity chlorine transporter) and *betB* (NAD^+^ dependent betaine aldehyde dehydrogenase), involved in synthesizing osmoprotectant glycine betaine in response to oxygen, chlorine, and osmotic stress [19], were also enriched in enteric strains. We also noted the absence of *nhaR*, the transcriptional activator of *nhaA* (a pH-dependent sodium-proton antiporter that responds to acidic conditions such as those prevailing in GI tract), in environmental and animal strains. All genes described above are associated with stress response and defense to multiple antimicrobials and stresses (e.g., acidic and osmotic stresses).

In addition to the stress defense systems, we found that the enteric strain-specific gene set included *hylE*, which encodes the pore-forming toxin involved in invasion of epithelial cells [20], [21]. A homologous gene is also found in other pathogenic strains such as *Shigella flexneri* and *Salmonella typhi* [22]. We also noted the absence of *sfmH*, a putative fimbrial-like adhesin protein, and two fimbrial-like proteins in environmental strains. Although the exact role of the *sfm* operon remains unclear, it shows sequence and organizational similarity to Type 1 fimbriae (proteins that mediate adhesion to intestinal epithelia and are commonly found in many Gram-negative bacteria) and possibly contributes to successful colonization of a range of surfaces, including epithelial cell surfaces [23]. Genes encoding proteins related to iron transport, heme-binding lipoproteins involved in utilization of multiple heme sources, and a maltose permease (*malF*) were also rarely present in environmental strains. High iron availability stimulates growth of enteric pathogens and their increased adhesion to the epithelial cells [24]. Disaccharide maltose metabolism supports the intestinal colonization of *E. coli* [25], [26].

Taken together, the absence of the stress defense and adherence-associated genes for evading host immune systems and attaching to epithelial tissues in the genomes of environmental strains indicates that the latter may not be as effective as enteric strains in colonizing the human GI tract.

### Validation of Microarray Performance used in this Study

Validating the performance of microarray experiment is essential for correct interpretation of hybridization data. We thus assessed the performance of oligonucleotide-spotted (spotted) microarrays used in this study based on hybridizations with selected, sequenced tester strains and compared it to the performance of the oligonucleotide, *in situ* synthesized microarrays used in our previous study, which represent an advanced microarray technology [27]. In brief, we identified the genes shared between the reference and the tester strains based on whole genome sequence comparisons and estimated the expected observed microarray hybridization signal based on the identity between the probe and the target gene in the tester strains using the BLAST score ratio approach, as described previously [27]. We found that probes gave signal comparable to the expected signal based on the sequence comparisons in 88.2% of the cases for tester strains showing about 95% gANI to the reference strain, resulting in 5.3% of false negatives (no microarray signal although gene is conserved in tester strain) and 6.5% false positives (microarray signal above background signal although the gene is absence in tester strain) (Fig. S1). These values were only slightly lower than those of the *in situ* synthesized microarrays (90.8%, 4.6%, and 5.3%, respectively). In addition, the performance of our microarrays was not dependent on the genetic divergence of the tester strains, for tester strains showing 93%–98% gANI to the reference strain (Fig. S1). Thus, the great majority of microarray probes provided reliable results and, despite several inherent limitations of microarray technology, our conclusions are conservative and robust.

## Discussion

Niche specialization often occurs by the acquisition of new traits that help organisms become better adapted to a new environment or an environmental fluctuation [28]. Specialization is also frequently accompanied by gene loss (also called reductive evolution), which is the result of losing genes that are no longer useful in the current niche [29]. Since *Escherichia* populations likely undergo biphasic lifestyle, i.e., periods of host-associated evolution followed by periods of host-independent evolution, we hypothesized that strains frequently recovered and persisting outside human hosts (environmentally-adapted) have undergone specialization and gain/loss of genes selected by their current/preferred habitat. To elucidate these genes, we compared environmental strains (G-III) against enteric strains (G-II), including MG1655, 042 (EAEC), and human fecal isolates. It should be mentioned that the environmental (or enteric) strains analyzed in this study might not be representative of the populations inhabiting the environmental habitats (or the GI tract) where the strains were isolated due to biases associated with cultivation. Nevertheless, the environmental and enteric strains showed clear phylogenetic separation (Fig. 1A, see also the discussion below on the effect of phylogeny) and several of the environmental and enteric strains have been isolated frequently from their (presumed) preferred habitats (e.g., C-III, C-IV, and C-V strains from a range of freshwater sources [11] and MG1655-lke strains from human fecal samples), indicating that they might represent well natural and enteric populations. In addition, the environmental and enteric groups compared here included representatives from five distinct phylogenetic lineages, i.e., G-III from C-III, C-VI, and C-V clades and G-II from C-I and *E. coli* clades and (Fig. 1). Therefore, we suggest that the results presented here represent a useful basis for future, more detailed investigations.

Comparative genomic hybridization revealed two sets of genes that are conserved in enteric strains but rarely found in environmental strains, indicating that these functions may be important for survival in the human host (Fig. 3). The gene sets encode i) stress response and defense systems to multiple drugs, acids, and DNA damage, and ii) adhesion to the human host tissues. A previous comparative microarray hybridization analysis of 45 *E. coli* isolates of environmental, human, and warm-blooded animal origin, which did not overlap with the strains used in our study, indicated that the major stress defense-associated genes were highly conserved in all isolates (oxidative/acidic/osmotic conditions, heat/cold shocks, toxic compounds, DNA protection/repair etc.) [12]. Our results confirmed that the majority of stress defense gene set was highly conserved among our twenty-seven strains. We thus suggest that the genes uniquely identified as specific or enriched in enteric strains in our study (e.g., *emrYK*, *evgAS*, *umuDC*, *nhaR,* and *betBT*) probably confer an improved fitness advantage that is specific for colonization on the human GI tract. Our results also revealed the loss of adherence-associated genes encoding hemolysin E (*hlyE*), fimbrial-like adhesin protein (*sfmH*), and nutrient uptake (iron and maltose) proteins in environmental strains. Bacterial adherence is necessary for the colonization of mucosal tissue surfaces for both pathogenic and commensal bacteria. A recent study revealed that the genes associated with bacterial adherence to the host proteins were significantly enriched in the human microbiome [30]. Thus, it is likely that the environmental strains may have decreased ability to successfully colonize epithelial cells compared to enteric strains. It will be interesting to quantitatively determine how essential these genes are for effectively colonizing the human mucosal surfaces and whether or not they could represent new virulence/colonization factors or therapeutic targets.

The patterns of gene content conservation between human and environmental strains (Fig. 1B) are fairly consistent with their phylogenetic groupings based on MLSA analysis (Fig. 1A), while a positive correlation (Pearson correlation = 0.75) of the gene content conservation with the degree of evolutionary relatedness was observed (Fig. 2). However, gene content patterns of some environmental strains (e.g., TW09231 and TW14182 belonging to G-I) were more similar to those of human strains than to environmental strains (G-III and G-IV), suggesting that phylogeny does not fully explain the gene content profiles or that these particular strains recently passed through a human host. For this reason, and also because phylogeny is apparently interlinked with the lifestyle/ecology of the isolates (i.e., more phylogenetically related isolates appear to originate from more similar habitats/hosts), we did not attempt to remove the effect of phylogenetic relatedness on the gene signatures observed between human vs. environmental strains.

Although DNA microarrays provide rapid and cost-effective means for genomotyping closely related organisms, this technology cannot distinguish whether genes identified as ‘not hybridized’ in tester strains are ‘truly absent’ or ‘present but sufficiently divergent to not provide hybridization signal above background signal’. Further, microarray-based approach cannot detect genes present in the tester strains that are not represented in the microarray platform. For instance, although the CFT073 strain (UPEC), a colonizer of the urinary tract, is negative for the hemolysin E, it can induce hemolysis by another pore-forming toxin, *α*-hemolysin (*hlyA*), which was not represented in our microarray. Accordingly, the possibility that the two gene sets found to be absent in environmental strains are actually present or evolutionarily divergent cannot be ruled out.

Whole genome sequencing approach sidesteps the limitations of microarray technology. By examining the whole genome sequence data of environmental *Escherichia* strains reported by Luo et al. [13], we observed that several of the human strain-specific genes (e.g., *emrY* and *betB*) found in our study are not present in the genomes of the environmental strains, although the strain collection used by the previous study is largely different from that of our study (e.g., our collection includes fifteen strains that have not been sequenced yet). Further, we also identified, consistently with the findings reported above (e.g., Fig. 3), that oxidative stress defense (e.g., glutaredoxin 2 and glutathione S-transferase), efflux pump (*ydjE*), adhesin (*aidA*), and maltose catabolism (*ycjT*) were conserved in the genome sequences of human but not environmental strains, which was not reported previously [13]. Our comparative genomic hybridization revealed that at least 1,863 genes are shared by all 27 strains used in our study (core genes; Figures 1 & 2). Previous studies based on whole genome sequencing or comparative microarray hybridization reported that 2,200, 1,976, and 993 genes are conserved in 17, 20, and 61 *Escherichia* strains, respectively [10], [31], [32]. Since the number of core genes decreases as more strains are added, our finding (1,863 core genes in 27 strains) is in a good agreement with the previous estimations, further validating the use of microarray for strain genomotyping in this study.

In summary, our results suggested that metabolic and regulatory genes and pathways map better to the preferred habitat of *Escherichia* organisms than phenotypic traits or 16S rRNA gene sequence data (all *Escherichia* isolates used in this study show <1% difference in their 16S rRNA gene sequences), and provided several biomarkers (e.g., *umuD* and genes encoding putative fimbrial-like and outer membrane receptor for iron transport proteins) distinguishing enteric strains from those of environmental and animal sources. Such biomarkers may constitute more reliable and better means for fecal contamination risk assessment compared to present approaches, which are based on phenotypic and culture-based techniques.

## Materials and Methods

### Strains and Growth Conditions

*Escherichia* strains were obtained from the ECOR *Escherichia coli* Reference Collection and the STEC Center at Michigan State University (shigatox.net). Our collection consisted of fifteen non-sequenced and uncharacterized strains and twelve strains whose genome sequences (complete or draft) were available from the National Center for Biotechnology Information website, ftp://ftp.ncbi.nih.gov/) and were included for comparison and validation purposes of the microarray results. All strains were grown and maintained in Luria Broth or on LB agar (Miller formulation) at 37°C with shaking when applicable. Table 1 provides the details about each strain used in the study.

**Table 1:** *Escherichia* strains used in this study.

Strain	Lineage	Source	Pathotype	Location isolated	Reference
Sakai	*E. coli*	Human	EHEC	MI, USA	[40]
EDL933	*E. coli*	Human	EHEC	MI, USA	[41]
MG1655	*E. coli*	Human	–	–	[42]
042	*E. coli*	Human	EAEC	Lima, Peru	[11]
e2348/69	*E. coli*	Human	EPEC	England	[43]
CFT073	*E. coli*	Human	UPEC	MD, USA	[44]
2457T	*S. flexneri*	Human	EIEC	Japan	[45]
*9194*	*E. albertii*	Human	Diarrheic	Bangladesh	[11]
*19982*	*E. albertii*	Human	Diarrheic	Bangladesh	[11]
TW11930	Clade I	Human	–	Guinea Bissau	[11]
TW11966	Clade I	Human	–	Guinea Bissau	[11]
TW12018	Clade I	Human	–	Guinea Bissau	www.shigatox.net
TW14421	Clade I	Human	–	Guinea Bissau	www.shigatox.net
TW09231	Clade III	Water	Avirulent	MI, USA	[13]
TW09254	Clade III	Water	–	MI, USA	[11]
TW09266	Clade III	Water	–	MI, USA	[11]
TW09276	Clade III	Water	Avirulent	MI, USA	[13]
TW11588	Clade IV	Soil	Avirulent	Puerto Rico	[13]
TW14182	Clade IV	Water	Avirulent	MI, USA	[13]
TW09308	Clade V	Water	Avirulent	MI, USA	[13]
TW14264	Clade V	Water	–	MI, USA	[11]
TW14265	Clade V	Water	–	MI, USA	[11]
TW14266	Clade V	Water	–	MI, USA	[11]
TW14267	Clade V	Water	–	MI, USA	[11]
TW14263	Clade V	Animal (Raccoon)	–	MI, USA	[11]
Z205	Clade V	Animal (Parrot)	–	–	[11]
RL325/96	Clade V	Animal (Dog)	–	–	[11]

### Evolutionary Relatedness using Multi-locus Sequence Analysis (MLSA) and Whole Genome Sequence

MLSA sequencing was performed as previously described [11]. Briefly, five housekeeping genes (*aspC, clpX, icdA, lysP*, and *mdh*) were used and the primers to amplify these loci were obtained from publicly available databases at Michigan State University (www.shigatox.net), the University College Cork (mlst.ucc.ie/mlst/dbs/Ecoli), or they were designed de novo to target new MLSA loci based on a recent analysis of *E. coli* MLSA gene candidates [33]. The same PCR conditions as the MLST protocol (www.shigatox.net/stec/mlst-new/mlst_pcr.html) for amplification were used.

The pair-wise evolutionary relatedness between the tester and the reference (Sakai) strain was measured based on the genome-aggregate average nucleotide identity (gANI). For strains Sakai, EDL933, MG1655, e2348/69, CFT073, 2457T, TW09231, TW09276, TW11588, TW14182, and TW09308, whose genomes sequences were available, the gANI values were calculated based on all reciprocally conserved best-match genes between the reference and the tester genomes (pair-wise), as previously described [14]. For strains with no genome sequence available, the gANI values were calculated based on the average sequence identity of the five housekeeping genes described above and used in MLSA analysis (mlsaANI). When the mlsaANI was compared with the gANI for the genome pairs with available genome sequences, a strong linear correlation (R^2^ = 0.96) was observed, suggesting that the mlsaANI is predictive of the gANI (Fig. S2), which is also consistent with previous results [34]. Thus, the gANI for the 16 non-sequenced genomes was calculated using the equation (gANI = 1.50×[mlsaANI] –49.7).

### DNA Microarray Fabrication

The *E. coli* probe set from Qiagen (Valencia, Calif.) was spotted on Corning glass slides at Michigan State University’ Research Technology and Support Facility (East Lansing, MI) and contained 2 copies of probes specific for 5,978 gene sequences from one commensal strain (MG1655) and two EHEC strains (Sakai and EDL933). 5,943 probes of the microarray were 70-mer oligonucleotides and 35 probes varied in size from 41–69 bp. The arrays also contained 384 spots representing 12 randomized spots, which were used as negative controls.

### DNA Preparation and Hybridization

Genomic DNA was prepared from 5 mL overnight cultures in LB medium using the Wizard Genomic DNA Purification Kit (Promega) according to manufacturer’s recommended protocols. Total DNA concentrations were measured using a NanoDrop ND-1000 UV-Vis spectrophotometer (NanoDrop Technologies, Wilmington, DE) and stored at –20°C. Ten micrograms of DNA was sheared using a Heat Systems Ultrasonics W-225 20-kHz, 200-W cup sonicator (Misonix, Farmingdale, NY) to generate sheared genomic DNA from 0.5 to 3 kb fragments. For each sample, 300 ng of sheared DNA was labeled with either Cy3 or Cy5 dye using the methods described by Wick et al. [35]. Equal amounts of tester and reference (Sakai) strain samples were co-hybridized to the arrays as described in [36] (competitive hybridization) and washed according to the manufacturer’s instructions for hybridization using coverslips. The Genepix 4000B array scanner (Axon Instruments, Union City, Calif.) was used to scan the arrays were scanned and probe intensities were retrieved using the associated Genepix 6.0 software.

### Evaluating the Performance of Microarray Experiments: Spotted vs. *in-situ* Arrays

To obtain the signal threshold for calling a gene present based on microarray signal, the following approach was used. The probe sequence identity against available genome sequences was used to determine (*in silico*) gene presence/absence, using a minimum cut-off of a BLAST-based 83% sequence identity (BINF_score, 1 = presence or 0 = absence), as suggested previously [27]. Gene presence/absence based on hybridization data was determined using the average log hybridization ratio (signal), or LHR, that corresponded to probes with exactly 83% sequence identity (LHR_83%_), as described previously [27]. Our previous studies demonstrated that i) the LHR_83%_ outperformed other frequently used metrics in calling gene presence/absence from hybridization data and ii) the result of the subtraction of LHR_83%_ from LHR_100%_ remained constant for tester strains varying in their genetic divergence to the reference strain (i.e., LHR_100%_ – LHR_83%_ = 0.45±0.15). For strains whose genome sequences were not available (and thus did not have probes available to calculate LHR_83%_), we estimated the LHR_83%_ indirectly, using a normalization factor that was determined based on the analysis of strains from which both genome sequences and hybridization data were available. More specifically, the LHR_83%_ for the former strains was calculated using the equation LHR_83%_ = LHR_100%_ – 0.45; where LHR_100%_ was estimated using the five house keeping gene sequences available for each strain and their corresponding hybridization data. The gene probes of the MG1655 did not match the genome of the reference strain (Sakai) used in the hybridizations (gene were specific to MG1655); thus, for these probes we were not able to apply the LHR approach. Instead, we used a signal-to-noise ratio (SNR) approach by subtracting negative control signals from the signal of the probes, as described previously [27], [37]. The LHR or SNR value obtained from each probe signal was converted into experimental binary scores (EXPL_score, 1 = presence or 0 = absence) using LHR_83% BSR_ or 2.0 of SNR as the cut-off signal for presence, respectively. Thus, gene presence/absence using EXPL_scores was applied to strains whose genomes have not been sequenced yet and BINF_scores to sequenced strains.

To evaluate the performance of microarray experiments and platforms, the experimentally determined gene callings (EXPL_scores) were compared with the bioinformatically determined gene callings (BINF_scores) for selected sequenced strains (Fig. S1). Genes were classified into three classes based on the combination of the EXPL_score and the BINF_score: good probes (GP, BINF_score = EXPL_score), false negatives (FN, BINF_score = 1 and EXPL_score = 0), and false positives (FP, BINF_score = 1 and EXPL_score = 0). The performance of different microarray platforms was compared based on the fraction of the three classes of probes: GP, FN, and FP.

### Microarray Data Accession Number

The raw microarray intensity data have been deposited in the GenBank Gene Expression Omnibus (GEO) database under accession number GSE39667.

## Supporting Information

Figure S1 **Evaluation of the performance of in-situ synthesized vs. spotted microarrays.** The percentage of good probes (GP), false-positive probes (FP), and false-negative probes (FN) are plotted against the gANI of the tester strain to the reference strain (Sakai). Two sets of tester strains were used, one set related at ∼97.5% gANI to the reference strain (strains MG1655, 2457T, e2348/69, and CFT073) and the other set related at ∼92.5% gANI (strains TW9231, TW9276, TW9308, TW11588, and TW14182). The GP, FP, and FN percentages of the in-situ synthesized microarray were obtained from our previous study [27]; the GP, FP, and FN percentages of the spotted microarray were calculated in the present study as described in the Materials and Methods section.
(TIFF)

Figure S2 **Estimation of genome-aggregate gANI based on MLSA data.** The gANI of the tester strains to the reference strain was plotted as a function of the average nucleotide sequence identity of the five MLSA genes (mlsaANI). Note the strong correlation between the two values, which suggests that the mlsaANI can reflect the gANI value.
(TIFF)

Table S1 **Genes differentially enriched between enteric (G-II) and environmental (G-III) strains.** These genes underlie the results shown in Figure 3.
(DOCX)
