# Supplementary figures and images for: Genomic Diversity of Escherichia Isolates from Diverse Habitats
Source: PLoS One. 2012 Oct 8;7(10):e47005. doi: 10.1371/journal.pone.0047005 (PMC3466228; doi:10.1371/journal.pone.0047005)

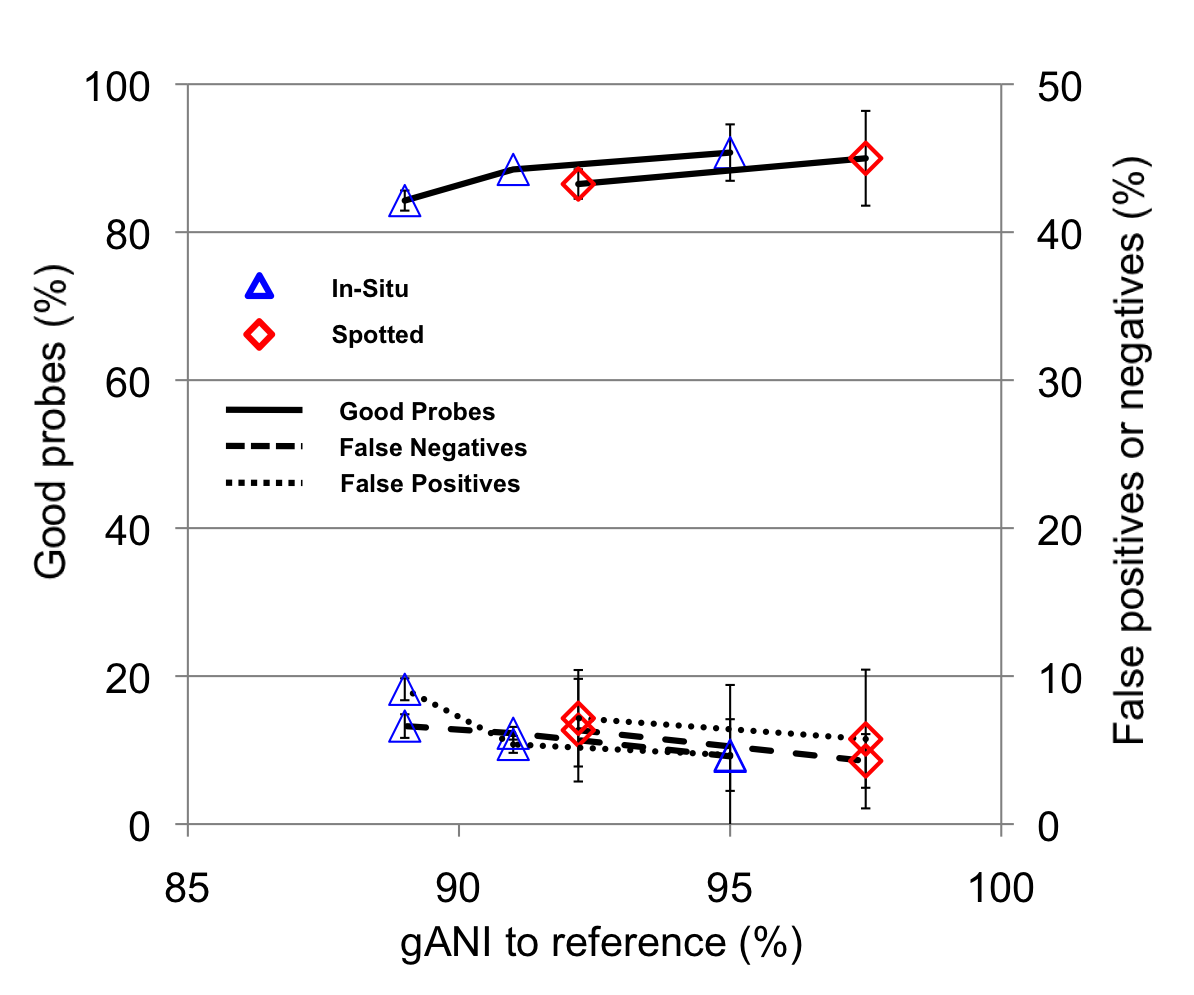

Supplement: Figure S1 — Evaluation of the performance of in-situ synthesized vs. spotted microarrays. The percentage of good probes (GP), false-positive probes (FP), and false-negative probes (FN) are plotted against the gANI of the tester strain to the reference strain (Sakai). Two sets of tester strains were used, one set related at ∼97.5% gANI to the reference strain (strains MG1655, 2457T, e2348/69, and CFT073) and the other set related at ∼92.5% gANI (strains TW9231, TW9276, TW9308, TW11588, and TW14182). The GP, FP, and FN percentages of the in-situ synthesized microarray were obtained from our previous study [27]; the GP, FP, and FN percentages of the spotted microarray were calculated in the present study as described in the Materials and Methods section. (TIFF) [file pone.0047005.s001.tiff]

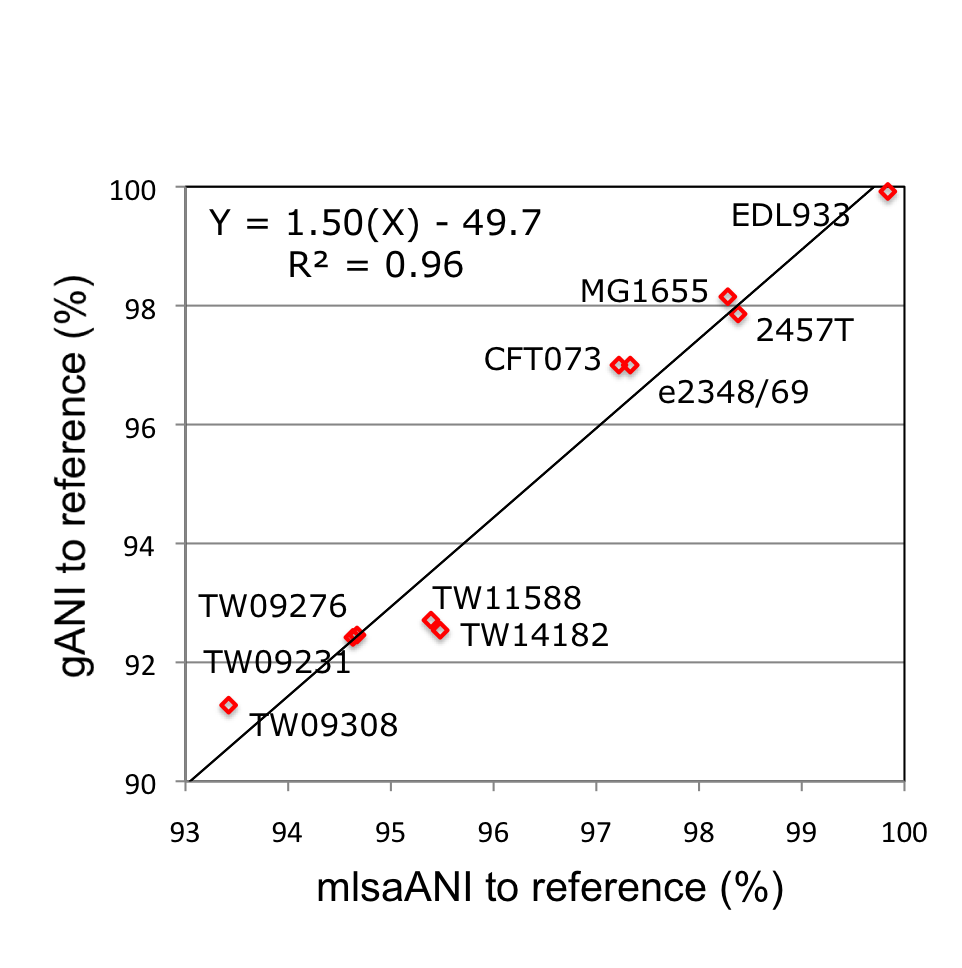

Supplement: Figure S2 — Estimation of genome-aggregate gANI based on MLSA data. The gANI of the tester strains to the reference strain was plotted as a function of the average nucleotide sequence identity of the five MLSA genes (mlsaANI). Note the strong correlation between the two values, which suggests that the mlsaANI can reflect the gANI value. (TIFF) [file pone.0047005.s002.tiff]
