# Supplementary material for: Genomic Diversity of Escherichia Isolates from Diverse Habitats
Source: PLoS One. 2012 Oct 8;7(10):e47005. doi: 10.1371/journal.pone.0047005 (PMC3466228; doi:10.1371/journal.pone.0047005)
Supplement: Table S1 — Genes differentially enriched between enteric (G-II) and environmental (G-III) strains. These genes underlie the results shown in Figure 3. (DOCX) [file pone.0047005.s003.docx]

| **Probe ID** | **Locus tag** | **Gene** | **Protein** |
| --- | --- | --- | --- |
| E100000020 | ECs0018 | nhaR | transcriptional activator of nhaA |
| E100000186 | ECs0188 | ldcC | lysine decarboxylase 2, constitutive |
| E100000210 | ECs0211/ECs0212 | yafT | putative aminopeptidase |
| E100000281 | ECs0319 | yagV | orf, hypothetical protein |
| E100000282 | ECs0320 | yagW | putative receptor |
| E100000283 | ECs0321 | yagX | putative enzyme |
| E100000284 | ECs0322 | yagY | orf, hypothetical protein |
| E100000285 | ECs0323 | yagZ | orf, hypothetical protein |
| E100000287 | ECs0326 | ykgL | orf, hypothetical protein |
| E100000301 | ECs0347 | - | orf, hypothetical protein |
| E100000304 | ECs0358 | betB | NAD+-dependent betaine aldehyde dehydrogenase |
| E100000306 | ECs0360 | betT | high-affinity choline transport |
| E100000317 | ECs0379 | yahK | putative oxidoreductase |
| E100000318 | ECs0380 | yahL | orf, hypothetical protein |
| E100000319 | ECs0380/ECs0382 | yahM | orf, hypothetical protein |
| E100000320 | ECs0382 | yahN | putative cytochrome subunit of dehydrogenase |
| E100000524 | ECs0595 | sfmH | predicted fimbrial-like adhesin protein |
| E100000635 | ECs0683 | ybeR | orf, hypothetical protein |
| E100000956 | ECs1138 | yccY | putative phosphatase |
| E100000957 | ECs1139 | yccZ | putative function in exopolysaccharide production |
| E100000958 | ECs1140 | ymcA | orf, hypothetical protein |
| E100000959 | ECs1141 | ymcB, ymcC | orf, hypothetical protein |
| E100000960 | ECs1142 | ymcC | putative regulator |
| E100000980 | ECs1252 | ycdG | putative transport protein |
| E100000983 | ECs1255 | - | putative acetyltransferase |
| E100000995 | ECs1267 | ycdP | orf, hypothetical protein |
| E100000999 | ECs1271 | ycdT | orf, hypothetical protein |
| E100001017 | ECs1422 | - | orf, hypothetical protein |
| E100001043 | ECs1448 | flgN | protein of flagellar biosynthesis |
| E100001045 | ECs1450 | flgA | flagellar biosynthesis; assembly of basal-body periplasmic P ring |
| E100001072 | ECs1477 | holB | DNA polymerase III, delta prime subunit |
| E100001151 | ECs1672 | ycgK | orf, hypothetical protein |
| E100001155 | ECs1677 | hlyE | hemolysin E |
| E100001156 | ECs1678 | umuD | SOS mutagenesis; error-prone repair; processed to UmuD'; forms complex with UmuC |
| E100001157 | ECs1679 | umuC | SOS mutagenesis and repair |
| E100001205 | ECs1736 | ychK | orf, hypothetical protein |
| E100001213 | ECs1742 | ychE | putative channel protein |
| E100001214 | ECs1743 | oppA | oligopeptide transport; periplasmic binding protein |
| E100001309 | ECs1922 | ydaJ | putative aminohydrolase (EC 3.5.1.14) |
| E100001379 | ECs2010 | - | probable enzyme |
| E100001382 | ECs2013 | ynbD | putative enzymes |
| E100001422 | ECs2055 | - | putative outer membrane receptor for iron transport |
| E100001430 | ECs0245 | - | orf, hypothetical protein |
| E100001441 | ECs2073 | - | orf, hypothetical protein |
| E100001442 | ECs2073 | - | putative glycoportein |
| E100001455 | ECs2088 | - | putative ATP-binding component of a transport system |
| E100001457 | ECs2090 | - | putative transport system permease protein |
| E100001458 | ECs2091 | - | putative hemin-binding lipoprotein |
| E100001459 | ECs2092 | - | orf, hypothetical protein |
| E100001468 | ECs2102 | - | putative enzyme |
| E100001469 | ECs2103 | - | putative sulfatase |
| E100001470 | ECs2104 | - | putative ARAC-type regulatory protein |
| E100001472 | ECs2106 | - | putative oxidoreductase, major subunit |
| E100001474 | ECs2108 | - | putative fimbrial-like protein |
| E100001475 | ECs2109 | - | putative fimbrial-like protein |
| E100001476 | ECs2110 | - | putative outer membrane protein |
| E100001482 | ECs2118 | ydeV | putative kinase |
| E100001486 | ECs2122 | ydeZ | putative transport system permease protein |
| E100001498 | ECs2134 | - | orf, hypothetical protein |
| E100001552 | ECs2288 |  | orf, hypothetical protein |
| E100001688 | ECs2427 | - | orf, hypothetical protein |
| E100001739 | ECs2480 | - | orf, hypothetical protein |
| E100001740 | ECs2481 | - | putative kinase |
| E100001741 | ECs2482 | - | putative aldolase |
| E100001744 | ECs2485 | - | putative oxidoreductase |
| E100001806 | ECs2548 | - | protein phosphatase 1 modulates phosphoproteins, signals protein misfolding |
| E100001856 | ECs2598 | cheA | sensory transducer kinase between chemo- signal receptors and CheB and CheY |
| E100001872 | ECs2612 | - | orf, hypothetical protein |
| E100001920 | ECs2694 | - | orf, hypothetical protein |
| E100001941 | ECs2777 | - | orf, hypothetical protein |
| E100001942 | ECs2777 | - | orf, hypothetical protein |
| E100002135 | ECs3068 | rtn | orf, hypothetical protein |
| E100002148 | ECs3081 | yejO | putative ATP-binding component of a transport system |
| E100002279 | ECs3205 | div | cell division protein |
| E100002324 | ECs3246 | emrY | multidrug resistance protein Y |
| E100002325 | ECs3247 | emrK | multidrug resistance protein K |
| E100002326 | ECs3248 | evgA | putative positive transcription regulator (sensor EvgS) |
| E100002327 | ECs3249 | evgS | putative sensor for regulator EvgA |
| E100002331 | ECs3254 | - | putative enzyme |
| E100002551 | ECs3467 | yfiN | orf, hypothetical protein |
| E100002794 | ECs3713 | - | orf, hypothetical protein |
| E100002795 | ECs3714 | ygeN | orf, hypothetical protein |
| E100002796 | ECs3715 | - | orf, hypothetical protein |
| E100002947 | ECs3897 | yqhG | orf, hypothetical protein |
| E100003059 | ECs4005 | yhaU | putative transport protein |
| E100003202 | ECs4145 | - | putative transferase |
| E100003411 | ECs4361 | yhiJ | orf, hypothetical protein |
| E100003508 | ECs4462 | yiaV | putative membrane protein |
| E100003675 | ECs4696 | yieO | putative transport protein |
| E100003783 | ECs4802 | - | putative aldose-1-epimerase (EC 5.1.3.3) |
| E100003925 | ECs5016 | malF | part of maltose permease, periplasmic |
| E100004212 | ECs5284 | - | orf, hypothetical protein |
| E100004413 | ECs1673 | ycgK | hypothetical protein |
| E100004648 | ECs0333 | slt | putative LysR-like transcriptional regulator |
| E100005325 | ECs2734 | atoE | putative DNA packaging protein of prophage CP-933R |
| E100005366 | ECs1847/ECs1849 | cysB - acnA | orf, Unknown function |
| E100005636 | ECs3712/ECs3713 | - | orf, Unknown function |
| E100005739 | ECs4350 | ylcC | putative phosphotransferase system enzyme subunit |
